# Applying a correction procedure to the prevalence estimates of overweight and obesity in the German part of the HBSC study

**DOI:** 10.1186/1756-0500-7-181

**Published:** 2014-03-27

**Authors:** Ute Ellert, Anna-Kristin Brettschneider, Susanna Wiegand, Bärbel-Maria Kurth

**Affiliations:** 1 Department of Epidemiology and Health Monitoring, Robert Koch Institute, General-Pape-Str. 62-66, 12101 Berlin, Germany; 2 Department of Pediatric Endocrinology and Diabetology, Charité Universitätsmedizin Berlin, Augustenburger Platz 1, 13353 Berlin, Germany

**Keywords:** Prevalence rates weight status, Self-reports, Corrected prevalence rates, Overweight, Body image

## Abstract

**Background:**

Prevalence rates for overweight and obesity based on self-reported height and weight are underestimated, whereas the prevalence rate for underweight is slightly overestimated. Therefore a correction is needed. Aim of this study is to apply correction procedures to the prevalence rates developed on basis of (self-reported and measured) data from the representative German National Health Interview and Examination Survey for Children and Adolescents (KiGGS) to (self-reported) data from the German Health Behaviour in School Aged Children (HBSC) study to determine whether correction leads to higher prevalence estimates of overweight and obesity as well as lower prevalence rates for underweight.

**Methods:**

BMI classifications based on self-reported and measured height and weight from a subsample of the KiGGS study (2,565 adolescents aged 11–15) were used to estimate two different correction formulas. The first and the second correction function are described. Furthermore, the both formulas were applied to the prevalence rates from the HBSC study (7,274 adolescents aged 11–15) which are based on self-reports collected via self-administered questionnaires.

**Results:**

After applying the first correction function to self-reported data of the HBSC study, the prevalence rates of overweight and obesity increased from 5.5% to 7.8% (compared to 10.4% in the KiGGS study) and 2.7% to 3.8% (compared to 7.8% in the KiGGS study), respectively, whereas the corrected prevalence rates of underweight and severe underweight decreased from 8.0% to 6.7% (compared to 5.7% in the KiGGS study) and from 5.5% to 3.3% (compared to 2.4% in the KiGGS study), respectively. Application of the second correction function, which additionally considers body image, led to further slight corrections with an increase of the prevalence rates for overweight to 7.9% and for obese to 3.9%.

**Conclusion:**

Subjective BMI can be used to determine the prevalence of overweight and obesity among children and adolescents. Where there is evidence of bias, the prevalence estimates should be corrected using conditional probabilities that link measured and subjectively assessed BMI from a representative validation study. These corrections may be improved further by considering body image as an additional influential factor.

## Background

For reasons of practicality and costs, many studies ask participants to state their height and weight (rather than measure them) to determine body mass index (BMI) [1,2]. There is considerable evidence in specialist literature to suggest that subjectively determined data on body mass index (BMI) shows distortions compared to objectively assessed BMI [3-12]. Examinations of this topic agree that subjective assessments tend to underestimate true BMI and that estimates of the prevalence of overweight and obesity consequently tend to be too low [3-12], whereas the prevalence rate for underweight is slightly overestimated [4,12]. The bias in the prevalence rates for under- and overweight based on self-reports is stronger in girls then in boys [4]. Furthermore, body image is an important factor which influences the distortions between subjectively and objectively assessed BMI [4,7,8].

To improve the prevalence estimates of weight status, the use of a correction formula is recommended [4,7]. A former publication [13] presented two procedures to correct subjectively assessed BMI using data from the German Health Interview and Examination Survey for Children and Adolescents (KiGGS) which provides both self-reported and measured height and weight from a representative subsample [14]. This procedure can be applied to studies of adolescents in Germany in which weight, height and body image are determined by self-report.

The necessity of implementing such a correction procedure becomes evident when comparing the prevalence of overweight and obesity estimates from the self-reported data of the German Health Behaviour in School Aged Children (HBSC) study [1,15] with the results of KiGGS, in which participants’ height and weight were measured in a standardized way. In KiGGS **10.4%** of children between 11 and 15 were overweight and **7.8%** obese, whereas the HBSC study reported that **5.5%** adolescents were overweight and **2.7%** obese. The difference in the prevalence rates for overweight between HBSC study and the KiGGS study was stronger in girls than in boys [16].

The aim of this study is to apply correction procedures developed previously to (self-reported) data from the HBSC study to determine whether the correction leads to higher, and consequently more realistic, prevalence estimates of underweight, overweight and obesity.

## Methods

### HBSC: study design and sample

The HBSC study is an international health study of children and adolescents sponsored by the World Health Organisation (WHO) and developed jointly in 1982 by scientists from Britain, Finland and Norway; since then it has been conducted every 4 years in a growing number of countries (41 in 2005/06) [1]. A consistent and structured procedure using a detailed research protocol must be followed to ensure international comparability. Both the methodology for compiling the samples and a core questionnaire with compulsory questions are pre-specified, but these can be supplemented by additional questions. The sampling begins at the level of schools and focuses on classes in the 5th, 7th and 9th grades. At least 1,500 students in each of the three age groups must be questioned per survey date and country to ensure a representative survey. The analysis presented here is based on the survey conducted in Germany in 2005–2006 (N = 7,274; 3,606 girls and 3,668 boys aged 11–15 years), in which the federal German states (*Bundesländer*) of Berlin, Hamburg, Hessen, North Rhine-Westphalia and Saxony took part [15].

### Measurement of BMI in the HBSC study

In the HBSC study all participants were asked to fill in a questionnaire in which they self-reported their height and weight. Subjective BMI was calculated using the formula BMI = weight/ height^2^.

Kromeyer-Hauschild’s BMI reference [17] values are currently used to define overweight and obesity in children and adolescents in Germany, in line with the recommendations of the Study Group on Obesity in Childhood and Adolescence (AGA) (see http://www.a-g-a.de). According to these reference values, children are considered overweight if they have a BMI above the 90^th^ age- and gender-specific percentile of the Kromeyer-Hauschild reference system. They are deemed to be obese if their BMI is above the 97^th^ percentile. Children or adolescents with a BMI below the age- and gender-specific 10^th^ percentile are defined as underweight; those below the third percentile are considered extremely underweight [17].

### Body image in the HBSC study

In the HBSC questionnaire all participants were asked to report their body image on a 5-point Likert-type scale. They were asked: "Do you think you are

➜ much too thin

➜ a bit too thin

➜ exactly the right weight

➜ a bit too fat

➜ much too fat ?" [1,13].

Responses were classified into the following categories: (1) ‘too thin’, (2) ‘right weight’, and (3) ‘too fat’.

#### The correction procedure

In the KiGGS study [14], randomly selected boys and girls aged between 11 and 17 years were asked to self-report their height and weight in face-to-face interviews at respective study centres before being measured and weighed in a standardized fashion. Trained staff measured body height without shoes to an accuracy of 0.1 cm using a portable Harpenden stadiometer (Holtain Ltd., UK). Body weight was measured to the nearest 0.1 kg, wearing underwear, using a calibrated electronic scale (SECA Ltd., Germany). BMI was calculated and classified as mentioned above [17]. Body image was measured with the official German translation which is also used in the German version of the HBSC study [1,13].

The correction methods suggested in [13] were applied to the prevalence estimates from the HBSC study. To correspond with the age groups included in the HBSC study, the representative KiGGS sample was restricted to adolescents aged between 11 and 15 years (N = 2,565; 1,216 girls and 1,349 boys) for the analyses presented here.

##### Correction procedure I

As explained in [13] the coefficients a_ij_ linking subjective and objective BMI classifications found on the basis of representative surveys are transferable to other studies based only on subjective statements, as long as those studies were carried out in the same age group of the same population in the same time period as the representative survey. Using correction formula (14) in [13] allows an estimation of the unknown true prevalence of overweight, normal weight and underweight, correcting for (gender-specific) distortions associated with subjective body image. Applying this to the HBSC study the estimated prevalence of the respective BMI class ${\hat{P}}_{i}^{\mathrm{HBSC}}$ will be

(I) $${\hat{P}}_{i}^{\mathrm{HBSC}}=\sum_{j=1}^{5}a_{\mathrm{ij}}^{\mathrm{KiGGS}}Q_{j}^{\mathrm{HBSC}}\;\left(i=1\ldots 5\right)$$

aijKiGGS=ΡΒΜΙKiGGS∈ΙiΒΜΙsubKiGGS∈Ιj

$${Q_{j}}^{\mathrm{HBSC}}=P\left(\mathrm{BM}I_{\mathrm{sub}}^{\mathrm{HBSC}}\in I_{j}\right)$$

Where a_ij_^KiGGS^ are the conditional probabilities calculated by the validation study KiGGS (as defined in formula (II) in [13]) and Q_j_^HBSC^ is the prevalence of subjective BMI category *I*_*j*_ found in the HBSC study.

##### Correction procedure II

In [13] a further correction formula (16) was introduced which can be applied for any study that has information not only on subjective BMI, but on body image as well. With data from KiGGS, it could be shown that the association between objectively and subjectively estimated BMI depends greatly on subjective body image. Adolescents who considered themselves “a bit too fat” or "much too fat" indicated a substantially lower subjective BMI on average than those who considered themselves “exactly the right weight” [13].

If a parallel validation study can provide estimates of the associations between objective and subjective BMI for different body image groups, this information can be used to further improve the prevalence estimates. This is the case for the HBSC study, using KiGGS as a corresponding validation study. For reasons of simplicity, the body image categories are combined into three groups as proposed and defined in [13]. Thus a further correction of the prevalence of BMI categories estimated by the HBSC study can be achieved by

(II) $${{\tilde{P}}_{i}}^{{}^{\mathrm{HBSC}}}=\sum_{k=1}^{3}\left[\sum_{j=1}^{5}\alpha_{\mathrm{ijk}}^{\mathrm{KiGGS}}Q_{\mathrm{jk}}^{\mathrm{HBSC}}\right]R_{k}^{\mathrm{HBSC}}\;\left(i=1\ldots 5\right),$$

where aijk=ΡΒΜΙKiGGS∈ΙiΒΜΙsubKiGGS∈Ιj,BIKiGGS=k

$$R_{k}^{\mathrm{HBSC}}=P\;\left({\mathrm{BI}}^{\mathrm{HBSC}}=k\right)$$

$R_{k}^{\mathrm{HBSC}}$is the prevalence of body image category k in the HBSC study, and QjkHBSC=ΡΒΜΙsubHBSC∈ΙjBIHBSC=k is the prevalence of subjective BMI category j in the group of adolescents with Body Image BI = k in the HBSC study.

## Results

### Preparation for correction procedure

KiGGS participants were classified as extremely underweight, underweight, overweight, obese or normal weight on the basis of both objectively measured BMI and subjective BMI. For HBSC participants, only the subjective BMI is available. In both studies, participants were asked to assess their body image. Tables 1 and 2 show the prevalence rates of BMI and body image categories among 11- to 15-year-olds according to KiGGS and the HBSC study.

**Table 1:** Prevalences of defined categories of measured BMI and subjective BMI among 11- to 15-year-old adolescents in Germany, stratified by gender in KiGGS and HBSC

**i =**	**All, %**			**Boys, %**			**Girls, %**		
	**P**_**i **_**KiGGS**	**Q**_**i **_**KiGGS**	**Q**_**i **_**HBSC**	**P**_**i **_**KiGGS**	**Q**_**i **_**KiGGS**	**Q**_**i **_**HBSC**	**P**_**i **_**KiGGS**	**Q**_**i **_**KiGGS**	**Q**_**i **_**HBSC**
1 = extremely underweight	2.4	3.6	5.5	2.6	4.5	5.5	2.1	2.7	5.5
2 = underweight	5.7	7.0	8.0	6.0	6.8	7.2	5.4	7.1	8.8
3 = normal weight	73.8	73.5	78.2	73.1	71.8	78.1	74.6	75.2	78.3
4 = overweight	10.4	9.4	5.5	10.7	10.5	6.2	10.0	8.3	4.8
5 = obese	7.8	6.5	2.7	7.6	6.4	3.0	7.9	6.7	2.5

P_i_: Measured Body mass index (i-th category).

Q_i_: Body mass index calculated from self-reported weight and height (i-th category).

**Table 2:** Prevalences of defined categories of Body Image among 11- to 15-year-old adolescents in Germany, stratified by gender in KiGGS and HBSC

**i =**	**All, %**		**Boys, %**		**Girls, %**	
	**R**_**i **_**KiGGS**	**R**_**i **_**HBSC**	**R**_**i **_**KiGGS**	**R**_**i **_**HBSC**	**R**_**i **_**KiGGS**	**R**_**i **_**HBSC**
1 and 2 = too thin	13.7	16.4	17.8	19.2	9.5	13.4
3 = right weight	43.0	44.2	47.2	49.6	38.6	38.6
4 and 5 = too fat	43.3	39.4	35.1	31.2	51.9	48.0

R_i_: Body Image (i-th category).

### For applying correction formula I

The conditional probabilities a_ij_^KiGGS^ required for formula (I) are presented in Table 3.

**Table 3:** BMI classification as a function of subjective BMI category among 11- to 15-year-old adolescents in Germany, stratified by gender in KiGGS

**a**_ij_^* KiGGS^	**BMI**_**sub**_^**KiGGS **^**ϵ**														
	**All**					**Boys**					**Girls**				
	**I**_**1**_	**I**_**2**_	**I**_**3**_	**I**_**4**_	**I**_**5**_	**I**_**1**_	**I**_**2**_	**I**_**3**_	**I**_**4**_	**I**_**5**_	**I**_**1**_	**I**_**2**_	**I**_**3**_	**I**_**4**_	**I**_**5**_
**BMI**^**KiGGS**^ ϵ															
**I**_**1**_	**44.0%**	8.6%	0.2%		0.5%	**34.3%**	12.3%	0.2%		0.9%	**60.9%**	5.0%	0.1%		
**I**_**2**_	26.8%	**36.5%**	2.9%			26.3%	**34.5%**	3.4%			27.7%	**38.6%**	2.5%		
**I**_**3**_	27.2%	54.8%	**91.2%**	15.2%	9.0%	36.3%	53.2%	**90.6%**	19.4%	11.6%	11.3%	56.5%	**91.8%**	9.7%	6.4%
**I**_**4**_	2.0%		5.2%	**60.5%**	12.2%	3.2%		5.5%	**54.0%**	15.4%			4.9%	**69.2%**	9.0%
**I**_**5**_			0.5%	24.3%	**78.3%**			0.3%	26.6%	**72.1%**			0.7%	21.1%	**84.6%**

*aijKiGGS=PBMI∈IiBMIsub∈Ij

BMI^KiGGS^: Body mass index.

BMI_sub_^KiGGS^: Body mass index calculated from self-reported weight and height.

I_1_ … I_5_: i-th category for BMI and j-th category for BMIsub (i.e., 1 = extremely underweight, 2 = underweight, 3 = normal weight, 4 = overweight, 5 = obese).

Using the a_ij_^KiGGS^ from Table 3 and the ${Q_{i}}^{{}^{\mathrm{HBSC}}}$ from Tables 1 and 2, the corrected prevalences of the five BMI categories are estimated from the HBSC study according to Formula (I). The corrected prevalence rates for both the whole study population and separately for boys and girls are shown in Table 4. In the following an example for calculation is shown:

$$\begin{array}{ll} P_{1}^{\mathrm{HBSC}} & =0.44*5.5+0.086*8.0+0.002*78.2\\ & \;+0*5.5+0.005*2.7\\ & =3.3 \end{array}$$

$$\begin{array}{ll} P_{2}^{\mathrm{HBSC}} & =0.268*5.5+0.365*8.0+0.029*78.2\\ & \;+0*5.5+0*2.7\\ & =6.7 \end{array}$$

$$\begin{array}{ll} P_{3}^{\mathrm{HBSC}} & =0.272*5.5+0.548*8.0+0.912*78.2\\ & \;+0.152*5.5+0.09*2.7\\ & =78.3 \end{array}$$

$$\begin{array}{ll} P_{4}^{\mathrm{HBSC}} & =0.02*5.5+0*8.0+0.052*78.2\\ & \;+0.605*5.5+0.122*2.7\\ & =7.8 \end{array}$$

$$\begin{array}{ll} P_{5}^{\mathrm{HBSC}} & =0*5.5+0*8.0+0.005*78.2+0.243*5.5\\ & \;+0.783*2.7\\ & =3.8 \end{array}$$

**Table 4:** Results of the BMI correction procedures, comparing the HBSC prevalences with the objective KiGGS prevalences among 11- to 15-year-old girls and boys in Germany

**i =**	**All, %**				**Boys, %**				**Girls, %**			
	**HBSC Q**_**i**_	**HBSC Corr1**	**HBSC Corr2**	**KiGGS P**_**i**_	**HBSC Q**_**i**_	**HBSC Corr1**	**HBSC Corr2**	**KiGGS P**_**i**_	**HBSC Q**_**i**_	**HBSC Corr1**	**HBSC Corr2**	**KiGGS P**_**i**_
1 = extremely underweight	5.5	3.3	3.2	2.4	5.5	3.0	3.0	2.6	5.5	3.9	3.5	2.1
2 = underweight	8.0	6.7	6.8	5.7	7.2	6.6	6.4	6.0	8.8	6.9	7.6	5.4
3 = normal weight	78.2	78.3	78.2	73.8	78.1	78.1	78.3	73.1	78.3	78.1	77.8	74.6
4 = overweight	5.5	7.8	7.9	10.4	6.2	8.3	8.3	10.7	4.8	7.4	7.4	10.0
5 = obese	2.7	3.8	3.9	7.8	3.0	4.0	4.1	7.6	2.5	3.7	3.7	7.9

P_i_: Measured Body mass index (i-th category).

Q_i_: Body mass index calculated from self-reported weight and height (i-th category).

Corr1: first correction.

Corr2: second correction.

### For applying correction formula II

Table 5 shows the objective BMI classification in KiGGS as a function of subjective BMI and subjective body image (a_ijk_^KiGGS^) for 11- to 15-year-old girls and boys in Germany.

**Table 5:** Objective BMI classification as a function of subjective BMI and body image among 11- to 15-year-old adolescents in Germany, stratified by gender in KiGGS

**a**_**ijK**_^KiGGS*^	**BMI**^**KiGGS**^_**sub**_** ϵ**																	
	**I**_**1**_	**I**_**2**_	**I**_**3**_	**I**_**4**_	**I**_**5**_	***Q***_***jk***_^***HBSC***^	**I**_**1**_	**I**_**2**_	**I**_**3**_	**I**_**4**_	**I**_**5**_	***Q***_***jk***_^***HBSC***^	**I**_**1**_	**I**_**2**_	**I**_**3**_	**I**_**4**_	**I**_**5**_	***Q***_***jk***_^***HBSC***^
	**All**						**Boys**						**Girls**					
**BMI**^**KiGGS**^ ϵ	$\bar{\mathrm{BI}}=1\left(\mathrm{too}\;\mathrm{thin}\right)$																	
**I**_**1**_	**59.6%**	15.4%	0.9%			*17.8%*	**46.1%**	16.1%	0.5%			*14.8%*	**76.1%**	14.5%	1.9%			*22.3%*
**I**_**2**_	22.7%	**42.3%**	16.9%			*22.0%*	25.6%	**44.0%**	14.8%			*16.4%*	19.2%	**39.8%**	21.9%			*30.4%*
**I**_**3**_	17.7%	42.3%	**82.2%**	100.0%	100.0%	*59.7%*	28.3%	39.9%	**84.6%**	100.0%		*68.0%*	4.7%	45.6%	**76.2%**	100.0%	1.8%	*47.3%*
**I**_**4**_						*0.4%*						*0.6%*						*0.0%*
**I**_**5**_						*0.1%*						*0.2%*						*0.0%*
$$\bar{\mathrm{BI}}=2\left(\mathrm{exactly}\;\mathrm{the}\;\mathrm{right}\;\mathrm{weight}\right)$$																		
**I**_**1**_	**20.5%**	4.2%	0.1%		9.2%	*5.2%*	**19.7%**	9.2%	0.2%		7.6%	5.0%	**23.2%**					*5.5%*
**I**_**2**_	39.5%	**34.1%**	1.6%			*8.5%*	31.5%	**26.8%**	1.3%			*7.7%*	68.3%	**40.2%**	1.9%			*9.5%*
**I**_**3**_	40.0%	61.8%	**96.6%**	49.1%	67.9%	*84.8%*	48.8%	64.1%	**96.0%**	59.0%	68.5%	*85.3%*	8.5%	59.8%	**97.3%**	28.5%	65.8%	*84.1%*
**I**_**4**_			1.8%	**44.1%**	22.9%	*1.3%*			2.5%	**30.8%**	19.4%	*1.9%*			0.9%	**71.5%**	34.2%	*0.6%*
**I**_**5**_			0.0%	6.8%		*0.2%*			0.0%	10.2%		*0.2%*						*0.3%*
$$\bar{\mathrm{BI}}=3\left(\mathrm{too}\;\mathrm{fat}\right)$$																		
**I**_**1**_	**25.5%**		0.1%			*1.1%*	**31.5%**		0.3%			*1.1%*						*1.1%*
**I**_**2**_		**19.6%**	0.3%			*1.7%*						*1.0%*		**26.4%**	0.5%			*2.1%*
**I**_**3**_	36.5%	80.4%	**87.4%**	8.2%	4.3%	*78.6%*	21.5%	100.0%	**84.3%**	9.0%	5.5%	*73.0%*	100.0%	73.6%	**89.2%**	7.2%	3.1%	*82.3%*
**I**_**4**_	38.0%		11.0%	**64.0%**	11.2%	*12.2%*	47.0%		14.6%	**59.9%**	15.3%	*16.0%*			8.9%	**68.9%**	7.2%	*9.6%*
**I**_**5**_			1.2%	27.8%	**84.5%**	*6.5%*			0.8%	31.1%	**79.2%**	*9.0%*			1.4%	23.9%	**89.7%**	*4.8%*

*aijKKiGGS=PBMIKiGGS∈IiBMIsubKiGGS∈Ij,BIKiGGS=k

BMI^KiGGS^: Body mass index.

BMI^KiGGS^_sub_: Body mass index calculated from self-reported weight and height.

I_1_ … I_5_: i-th category for BMI and j-th category for BMI_sub_ (i.e., 1 = extremely underweight, 2 = underweight, 3 = normal weight, 4 = overweight, 5 = obese).

BI^KiGGS^: Body image.

Qj_k_^HBSC^ : Prevalences of the subjective BMI categories j in the group of people with Body Image BI = k in HBSC.

An example of the calculation of corrected prevalences of the five BMI categories estimated from the HBSC study according to Formula (II) is mentioned below. In Table 4 corrected prevalence rates for both the whole study population and separately for boys and girls are shown.

$$\begin{array}{ll} P_{1} & =0.164*\left(0.596*17.8+0.154*22.0+0.009\right)\\ & \;\left(*59.7+0*0.4+0*0.1\right)\\ & \;+0.442*\left(0.205*5.2+0.042*8.5+0.001\right)\\ & \;\left(*84.8+0*1.3+0.092*0.2\right)\\ & \;+0.394*\left(0.255*1.1+0.042*1.7+0.001\right)\\ & \;\left(*78.6+0*12.2+0*6.5\right)\\ & =3.2 \end{array}$$

$$\begin{array}{ll} P_{2} & =0.164*\left(0.227*17.8+0.423*22.0+0.169\right)\\ & \;\left(*59.7+0*0.4+0*0.1\right)\\ & \;+0.442*\left(0.395*5.2+0.341*8.5+0.016\right)\\ & \;\left(*84.8+0*1.3+0*0.2\right)\\ & \;+0.394*\left(0*1.1+0.196*1.7+0.003*78.6\right)\\ & \;\left(+0*12.2+0*6.5\right)\\ & =6.8 \end{array}$$

$$\begin{array}{ll} P_{3} & =0.164*\left(0.177*17.8+0.423*22.0+0.822\right)\\ & \;\left(*59.7+1*0.4+1*0.1\right)\\ & \;+0.442*\left(0.4*5.2+0.618*8.5+0.966*84.8\right)\\ & \;\left(+0.491*1.3+0.679*0.2\right)\\ & \;+0.394*\left(0.365*1.1+0.804*1.7+0.874\right)\\ & \;\left(*78.6+0.082*12.2+0.043*6.5\right)\\ & =78.2 \end{array}$$

$$\begin{array}{ll} P_{4} & =0.442*\left(0*5.2+0*8.5+0.018*84.8+0.441\right)\\ & \;\left(*1.3+0.229*0.2\right)\\ & \;+0.394*\left(0.38*1.1+0*1.7+0.11*78.6\right)\\ & \;\left(+0.64*12.2+0.112*6.5\right)\\ & =7.9 \end{array}$$

$$\begin{array}{ll} P_{5} & =0.442*\left(0*5.2+0*8.5+0*84.8+0.068\right)\\ & \;\left(*1.3+0*0.2\right)\\ & \;+0.394*\left(0*1.1+0*1.7+0.012*78.6\right)\\ & \;\left(+0.278*12.2+0.845*6.5\right)\\ & =3.9 \end{array}$$

### Comparison of corrected with uncorrected prevalence rates

Table 4 summarizes the results of the two correction procedures both for all and separately for boys and girls. For comparison the uncorrected prevalence rates from HBSC and the true BMI from KiGGS are displayed as well. The first correction step substantially increases the prevalence of overweight and obesity and reduces the prevalence of underweight and severe underweight in the HBSC study. The prevalence rates of overweight and obesity increased from 5.5% to 7.8% (compared to 10.4% in the KiGGS study) and 2.7% to 3.8% (compared to 7.8% in the KiGGS study), respectively, whereas the corrected prevalence rates of underweight and severe underweight decreased from 8.0% to 6.7% (compared to 5.7% in the KiGGS study) and from 5.5% to 3.3% (compared to 2.4% in the KiGGS study), respectively. For boys the correction procedure led to more accurate estimates compared to girls. The second correction step, involving body image, led to further slight corrections in the same directions. The prevalence rates for overweight increased to 7.9% and for obese to 3.9%.

## Discussion

The present study shows that the correction of subjectively estimated BMI with a correction formula developed on the basis of the representative KiGGS sample leads to more accurate prevalence rates for overweight and obesity. After the correction procedure the prevalence rates in the HBSC study improved for overweight from 5.5% to 7.9% (compared to 10.4% in the KiGGS study), for obese from 2.7% to 3.9% (compared to 7.8% in the KiGGS study).

On the basis of the HBSC study in Wales, in which measurements were made in parallel with the questionnaire [5], the authors point out that measured data are definitely needed in order to assess the magnitude of misclassification. A study of Greek school students came to the same conclusion [10]. It is recommended that the degree of misclassification in subgroups of children and adolescents be examined more closely by a validation study. Due to its big sample size and the wide age range covered (11–17 years) the KiGGS study fulfils this role in Germany.

By carrying out parallel height and weight measurements in a sub-sample of the 11- to 17-year-old KiGGS study participants, it was possible to quantify the deviations between measured values and subjective statements in various subgroups. These data were used to develop a correction procedure for studies that record only subjective statements on height, weight and body self-image [13]. The correction method developed here on the basis of KiGGS data is applied in this report to the results of the HBSC study. In a previous study from Wick et al. [18], the same correction procedure was applied to another study that recorded subjective statements on height, weight and body self-image. In both cases, the prevalence rates of overweight and obesity were revised upwards and became more realistic, although not as much as would bring them in line with the KiGGS prevalence data. Jansen et al. [7] applied two different formulas, based on a multiple linear regression, to correct BMI derived from self-reports of 12- to 13-year old adolescents in the Netherlands. One included socio-demographic characteristics (country of origin and level of education) alone, the second one additionally contained body image. Both formulas led to higher prevalence estimates of overweight [7]. Wick et al. [18] as well as Jansen et al. [7] came to the same conclusion as this study: the formula considering body image led to more accurate estimates of overweight.

We analysed data from the HBSC 2005/2006 Survey, and not from the current HBSC survey, since the data from the KiGGS study were collected 2003–2006, almost the same time period as the earlier study. The true prevalence rates of overweight and obesity might differ or change over time, which also influences the self-reported weight bias [19]. Since the strongest underestimation is seen among overweight and obese adolescents, the appliance of a correction procedure is limited. It is recommended to collect measurements from a subsample to update the correction procedure. An update will be possible with the next waves of the KiGGS study.

The remaining differences between the KiGGS data and the corrected HSBC data may be explained by examining differences in methodology: The subjective statements on height and weight correlate with the subsequently measured values better in KiGGS than in other studies. The deviations between subjective and objective BMI among adolescents of normal weight are minimal; in the case of overweight and obese, however, there is a tendency for subjective BMI to underestimate measured BMI. Here, a special situation should be taken into consideration: while they were being questioned in the study centres, KiGGS participants were sitting face-to-face with the interviewer, and they were informed via an invitation letter that their height and weight would be measured as part of the examination programme. Thus, the participants were perhaps motivated to report more accurately than in an anonymous, written interview conducted in the context of their school classroom (as was the case in the HBSC study).

For boys the correction procedure led to more accurate estimates compared to girls. This might be due to the fact that the difference between prevalence estimates based on self-reports to prevalence rates based on measured data was already smaller for boys before the estimates were corrected. The stronger misreporting in girls may be associated with the desire to be leaner. Social desirability and social norms for thinness from which girls are stronger affected than boys might lead to a systematic underreporting of BMI in girls [3,4,7,11].

Differences between the two studies can also be seen according to body image; these were most distinct in the group who considered themselves "too fat" or "much too fat". Examining subjective BMI among KiGGS participants, two thirds of this group were normal weight and less than a third were overweight or obese. According to the HBSC study, more than three quarters had normal weight and only 19% were overweight or obese. These differences were much greater among girls than among boys [20,21].

## Conclusions

BMI based on subjectively assessed height and weight can certainly be used to estimate the distribution of overweight and obesity among children and adolescents. Biased prevalence estimates can be corrected if a representative validation study exists, from which the associations between measured and subjectively assessed BMI can be determined. These corrections are further improved by additional consideration of adolescents’ body image. We have shown here that coefficients linking categories of measured BMI with subjective BMI as well as body image in the KiGGS study can be used to correct the BMI categorization in the HBSC study that relied on subjective assessment. Analogue procedures are applicable to all national components of the international HBSC study. Provided that one considers smaller parallel validation studies requiring comparatively little effort when planning further assessments, the problem of the prevalence of overweight and obesity can be better characterized.

## Abbreviations

BMI: Body mass index; HBSC: German Health Behaviour in School Aged Children study; KiGGS: German National Health Interview and Examination Survey for Children and Adolescents; WHO: World Health Organisation.

## Competing interests

The authors declare that they have no competing interests.

## Authors’ contributions

UE and BMK were involved in the design and data assessment and BMK is the principal investigator of KiGGS. UE and BMK performed the statistical analyses, interpreted the results and wrote the manuscript. AKB was involved in writing the manuscript and made substantial contributions. AKB and SW revised the manuscript critically. All authors have read and approved the final version.
